# Modulating spontaneous brain activity using repetitive transcranial magnetic stimulation

**DOI:** 10.1186/1471-2202-11-145

**Published:** 2010-11-10

**Authors:** Ysbrand D van der Werf, Ernesto J Sanz-Arigita, Sanne Menning, Odile A van den Heuvel

**Affiliations:** 1 Sleep and Cognition, Netherlands Institute for Neurosciences, an institute of the Royal Netherlands Academy of Arts and Sciences, Meibergdreef 47, 1105BA Amsterdam, The Netherlands; 2 Clinical Neurophysiology, VU University medical center, PO Box 7057 1007MB Amsterdam, The Netherlands; 3 Dept Anatomy and Neurosciences, VU University medical center, PO Box 7057 1007MB Amsterdam, The Netherlands; 4 Radiology, VU University medical center, PO Box 7057 1007MB Amsterdam, The Netherlands; 5 Dept Radiology, CITA-Alzheimer Foundation, Parque Tecnológico de San Sebastián, P º Mikeletegi 61, 20009 Donostia-San Sebastián, Spain; 6 Dept Psychosocial Research and Epidemiology, Netherlands Cancer Institute, Plesmanlaan 121, 1066CX Amsterdam, The Netherlands; 7 Psychiatry, VU University medical center, PO Box 7057 1007MB Amsterdam, The Netherlands

## Abstract

**Background:**

When no specific stimulus or task is presented, spontaneous fluctuations in brain activity occur. Brain regions showing such coherent fluctuations are thought to form organized networks known as 'resting-state' networks, a main representation of which is the default mode network. Spontaneous brain activity shows abnormalities in several neurological and psychiatric diseases that may reflect disturbances of ongoing thought processes. Information about the degree to which such spontaneous brain activity can be modulated may prove helpful in the development of treatment options. We investigated the effect of offline low-frequency rTMS on spontaneous neural activity, as measured with fMRI, using a sequential independent-component-analysis and regression approach to investigate local changes within the default mode network.

**Results:**

We show that rTMS applied over the left dorsolateral prefrontal cortex results in distal changes of neural activity, relative to the site of stimulation, and that these changes depend on the patterns of brain network activity during 'resting-state'.

**Conclusions:**

Whereas the proximal changes may reflect the off-line effect of direct stimulation of neural elements, the distal changes likely reflect modulation of functional connectivity.

## Background

Brain activity underlying unconstrained thought can be visualized as resting-state networks[1-3]. Resting-state networks in health and disease are the topic of intensive investigation, but as yet little is known about factors that affect their appearance. The most well-known resting-state network, the default mode network (DMN) consists of concurrent activation of the medial prefrontal, the medial parietal and lateral parietal areas, in combination with medial and lateral temporal cortices[4]. This activity shows systematic deactivations during cognitive task performance that appear to be task-relevant[5,6]. Other functions ascribed to the DMN include introspection, memory processes and mind-wandering, although part of the activity is also accounted for by non-cognitive functions. It is not well known if the areas involved in the DMN contribute to a unified and general function or whether they represent separate contributions to ongoing thought. The fluctuating activity of the DMN seems to be controlled at least in part by different networks and regions[7,8]. The fluctuations of the DMN and other resting state networks occur both spontaneously and in relation to mental activity. It is as yet little known inhowfar external stimulation of the brain changes the resting state activity. Repetitive Transcranial Magnetic Stimulation (rTMS) is a tool to non-invasively and painlessly stimulate the brain[9]; depending on the stimulation parameters, the effect of a train of pulses will either facilitate or inhibit the activity of a neural ensemble[10,11]. The effects of rTMS outlast the period of stimulation and, depending on the parameters used, may last up to approximately half an hour or longer. Such stimulation has been shown to result in robust changes in various aspects of brain functioning, such as neuroransmitter release, task-related brain activity, motor output and behavioural indices [12-16]. Stimulation over the dorsolateral prefrontal cortex is an often used and potent modulator of both brain activity and task performance[17-23]. To our knowledge, however, no studies have as yet investigated the effect of dorsolateral rTMS on resting state brain activity.

We here use a single session of low-frequency rTMS treatment on the dorsal lateral prefrontal cortex to study resting state brain activity in healthy subjects. We hypothesized that rTMS, applied locally over the left dorsal lateral prefrontal cortex, would alter the strength or the spatial distribution, or both, of spontaneous brain activity. We analysed the effects of the rTMS treatment on the most well-known of the resting state brain networks, the default mode network. This network is the most robust of the resting state networks under task-free conditions. It is especially relevant for psychiatric disorders, especially depression, in which dorsal lateral rTMS appears effective[24].

We compared the stimulation with a sham stimulation to control for peripheral effects of stimulation and the placebo effects of the treatment; the sham condition consisted of tilting the coil 90 degerees, such that it rested on the head with its edge. The bone conduction of the clicking sound would then be comparable to the real stimulation. We took care to blind the subjects by not showing the angulation of the coil and explaining that the sensation of stimulation could vary from session to session. The choice of a placebo condition is notoriously hard in rTMS research since treatment of 'inactive areas' to show specificity of the stimulation may sometimes lead to unblinding of the subjects or to unwanted effects due to passive or active spreading of the activity (e.g. [25]).

## Results

### Resting motor thresholds

We determined resting motor thresholds in our subjects on both days of testing. No within-subject differences between thresholds were found between conditions, resulting in stimulation intensities (at 90% of resting motor threshold) that were not different between conditions.

### RSN networks

**Figure 1:**
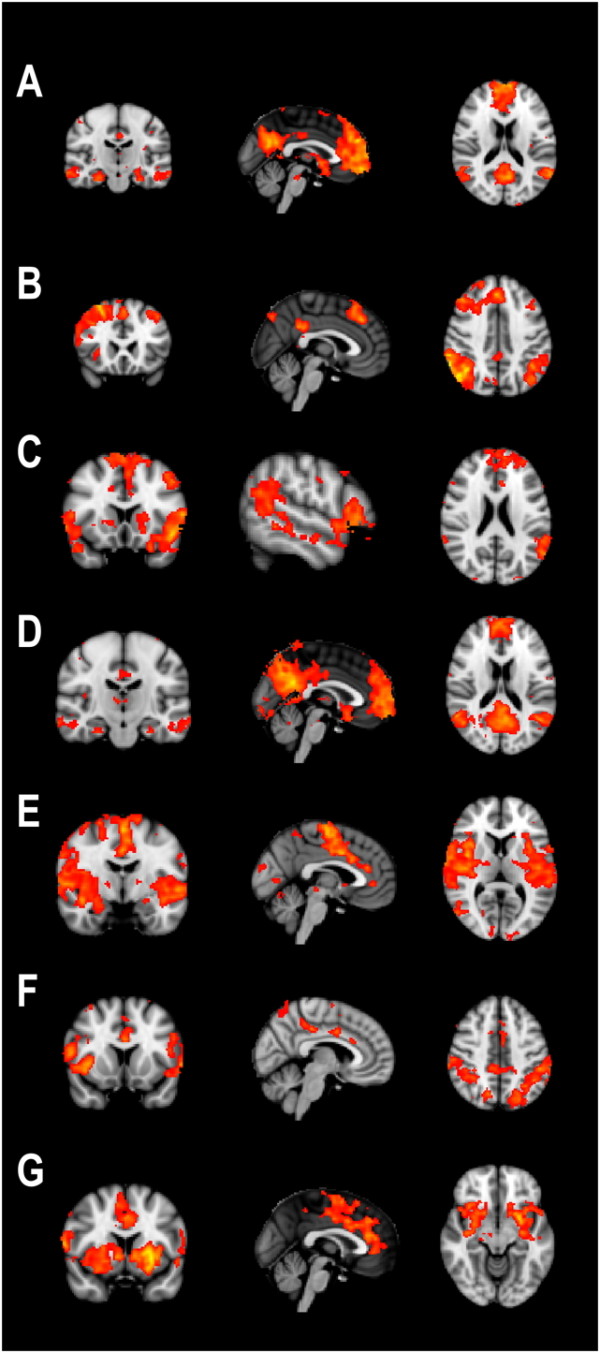
**Meaningful components from the independent component analysis across the 20 resting state acquisitions**. The topmost component represents the DMN used for this analysis. Note that component D also represents the DMN, but slightly more posteriorly weighted.

Our analysis yielded 41 resting-state networks. Of these networks, we selected the meaningful components that corresponded to networks described earlier, i.e. the 7 components shown in figure 1[4,26]. We did not find an RSN corresponding to a 'working memory network' in the left hemisphere in this study [26], although we did observe its right-hemisphere homologue.

**Figure 2:**
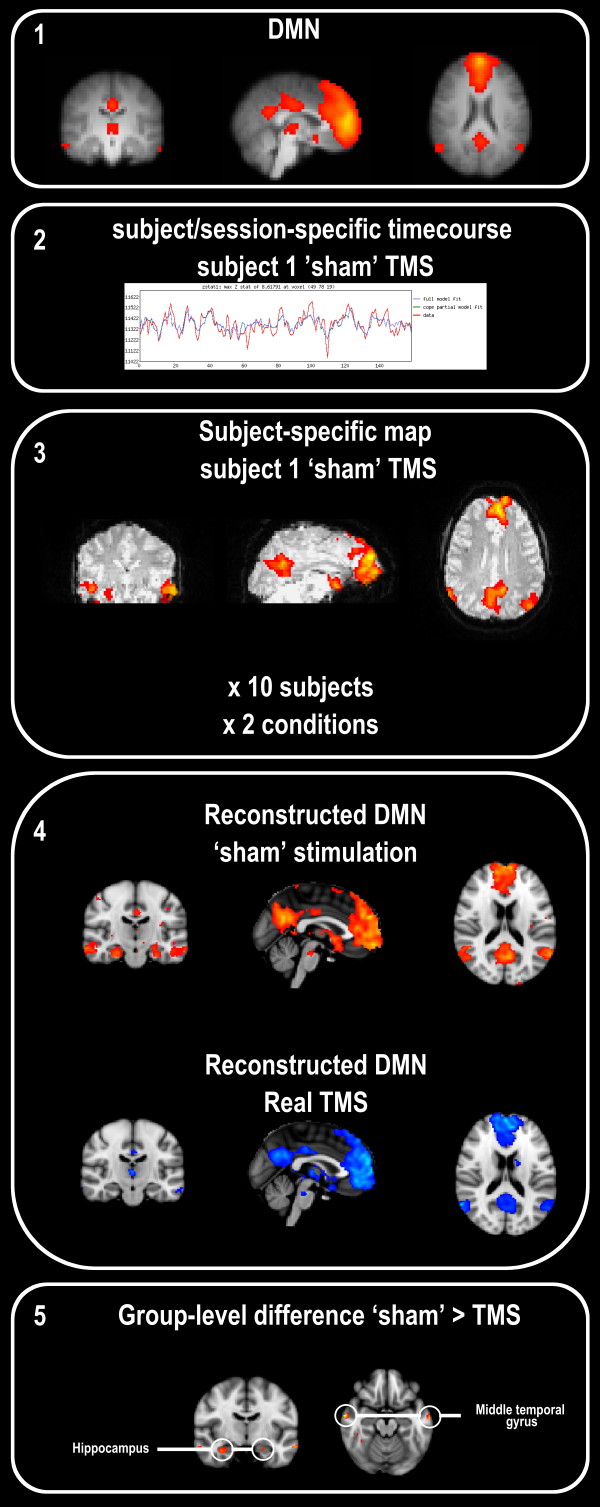
**Step-wise procedure and end result of the regression method**. From the independent component representing the DMN, obtained with spatiotemporal group ICA (1), we used the subject-and-session specific timecourses (2) to reconstruct subject-specific 3D maps for the 10 subjects*2 sessions (3). As a verification, we calculated the group means of the sham and rTMS groups separately ('reconstructed IC4') and observed that the DMN-characteristic pattern of regional co-activations occurred in both groups, with an additional co-activation of the hippocampal and lateral temporal cortices after sham stimulation but not real stimulation (4). Upon formal testing, using groupwise within-subject component general linear modelling, the lateral temporal regions differed significantly between conditions, such that their activation was reduced after rTMS. The image is thresholded at z > 2.3, showing the extent of the reductions and subthreshold reductions in the bilateral hippocampus (5). The reverse contrast showed an increase in activation of the right caudate nucleus (not shown).

For this analysis, we focused on the topmost component, representing the DMN. Our results indicate that this component comprises the well-known regions, i.e. the medial prefrontal cortex, precuneus and lateral parietal cortex. In accord with several studies, IC4 also encompassed the hippocampus proper and middle temporal gyrus (Figure 2). We reconstructed the groupwise means (i.e. sham and rTMS conditions: n = 10 each) and observed the same network consisting of the same regions for the two stimulation conditions separately, with the notable exception of the hippocampus and lateral temporal cortex that were not observed in the low-frequency rTMS condition (Figure 2). Upon formal testing, contrasting condition using session as a within-subject factor, we observed that the activations in the lateral temporal cortex were significantly stronger after 'sham' rTMS (z > 3.1; Table 1). Lowering the significance threshold to z > 2.3 in the regions that are part of the DMN in this analysis, the hippocampus proper showed reduced activation bilaterally (figure 1). In the reverse contrast, activation in the right caudate nucleus was seen after rTMS but not sham (z > 3.1; Table 1).

**Table 1:** Brain regions showing reduced or increased activity after rTMS.

Contrast	Zmax	x	y	z	Area
sham > rTMS	3.23	-64	-8	-16	Left middle temporal gyrus
	3.54	62	-6	-18	right middle temporal gyrus
sham > rTMS	3.28	14	16	8	Right caudate nucleus

The left and right middle temporal gyrus appeared at z > 3.1, using a search mask consisting of the distribution of the original component.

## Discussion

We here show changes of DMN activity upon treatment with inhibitory low-frequency rTMS intervention. The alterations in DMN strength occurred distal to the site of stimulation, i.e. prefrontal stimulation led to reductions in DMN activity bilaterally in the temporal lobes and an increase in the caudate nucleus. Previous reports have shown the existence of multiple constellations of simultaneously active areas in the human brain, that supposedly correspond to specific brain functions or processes [26]. The default mode network is the most well-known of these and can also be observed as inactivations during task performance, measured using BOLD fMRI or PET [4]. The DMN may correspond in part to introspection, reflection or spontaneous cognition in the absence of external stimulation, but also reflects processes unrelated to conscious cognition such as maintenance of functional integrity of brain networks or metabolic demands[27,28]. Several reports have shown that the hippocampal formation and adjacent medial temporal cortical structures are part of the DMN [29-31]. These areas are well-known for their role in semantic and episodic memory formation and retrieval as well as novelty detection.

We analysed the effects of rTMS treatment on the activity of the most important resting state network. We performed a within-component comparison of the two conditions, aimed at revealing differences at the level of the DMN. A different approach would be to investigate interactions between networks and their timing[32]. Such studies would allow to investigate the balance between different brain systems and the changes that rTMS may exert on such interactions. A limitation of this study is that the coil was placed over the left dorsolateral prefrontal cortex using the motor 'hotspot' as a landmark. Such a coil placement technique is more variable with respect to the underlying brain structures targeted than neuronavigation approaches using the individual MRI of the subjects[33]. On the other hand, the results presented here would be an underestimation, if anything, of the effect that more targeted interventions could have. At the same time, coil placement using head-based landmarks does make the technique more readily available outside of a specialized laboratory setting, and more easy to implement should the technique become a treatment option.

Repetitive TMS as a treatment may be relevant for psychiatric disorders such as depression, obsessive compulsive disorder or schizophrenia that are characterized by spontaneous intrusive thoughts. Indeed, several psychiatric disorders are characterized by abnormal brain activity in the resting state[34-37]. It would be of interest to modulate ongoing brain activity, offering either direct clinical benefit or a window of time in which a patient might be more receptive to other kinds of therapy, such as psychotherapy. Indeed, rTMS has been considered as a treatment option, most notably in major depression[24,38]. Since firstly, stimulating the dorsal lateral prefrontal cortex appears to benefit mood disturbances; and secondly, depression is associated with disturbed DMN activity, this raises the question of whether mood improvements after rTMS are associated with changes in the DMN, even though the area stimulated lies outside the DMN itself. Speculatively, the reduced activation in hippocampal and lateral temporal areas after rTMS over the dorsolateral prefrontal cortex may affect autobiographical or semantic memory retrieval, of importance for disturbed self-referential cognitive tendencies in psychiatric disease: depressed patients observing and re-appraising negative images, for example, showed a failure to suppress DMN activity in lateral temporal cortices, while even increasing activity in the medial temporal lobe [36]. Suppression of temporal lobe activity may thus underlie the beneficial effects of prefrontal rTMS on depressed mood, through an effect on spontaneous mental activity outlasting the duration of treatment.

## Conclusions

Low-frequency rTMS over the left dorsolateral prefrontal cortex affects off-line resting-state brain activation. The intervention reduces RSN activity within the DMN. The reductions of activation occur in the temporal lobes that are distal from the area stimulated, suggesting an effect of rTMS on long-range functional connectivity.

## Methods

Ten right-handed healthy controls (6 female; mean age 25.5 years) were entered in a cross-over design: two single session treatments (counterbalanced across the group) of low-frequency (1 Hz) rTMS versus sham for 20 minutes on the left dorsolateral prefrontal cortex. The coil was placed 5 cm anterior to the 'motor hotspot', i.e. the location where stimulation led to maximal motor responses in the contralateral hand. We determined the resting motor threshold at this 'motor hotspot' as that intensity at which 5 visible hand/finger responses could be evoked out of a series of 10 consecutive stimulations.

Repetitive TMS was applied using a hand-held figure-of-eight TMS coil (Medtronic MagOption). Directly following the off-line rTMS treatment, 160 volumes were acquired of the brain in 'resting-state' with a 3T Philips Intera MRI (EPI, TR 2.30 sec., TE 30 msec., matrix 96×96 pixels, field of view 220×220 mm, flip angle 80°, 35 slices, slice thickness 3 mm, in plane resolution 2.3×2.3 mm).

Imaging data were first converted from the original PAR/REC files to Analyze format using MRIcro (Chris Rorden). We then used pre-processing and statistics using tools implemented in FMRIB's Software Library (FSL, http://www.fmrib.ox.ac.uk/fsl) as follows: the functional MR images were motion-corrected using MCFLIRT and nonbrain tissue was removed with BET. The images were spatially smoothed using a Gaussian kernel of six mm full-width-at-half-maximum (FWHM) and a high-pass temporal filtering was applied (Gaussian-weighted least-squares straight line fitting, with sigma = 100 s). The functional scan was then aligned to the subject's high resolution T1-weighted image, and subsequently to the MNI152 standard through affine linear registration as implemented in FLIRT.

After preprocessing, a unique 4D data set was created by concatenating all the individual data. This concatenated fMRI data set was decomposed using ICA as part of Multivariate Exploratory Linear Optimized Decomposition into Independent Components (MELODIC) to identify homogeneous patterns of brain activity in our subjects[39]. The analysis used automatic estimation of dimensionality to control the number of components reported. We extracted the top meaningful components ranked according to the amount of explained variance, each representing statistically independent resting state networks. Components were deemed 'meaningful' on the basis of visual inspection of the spectra and spatial distribution: e.g. networks consisting of artefacts such as ventricular, white matter and brain circumferential activations were excluded, as were components showing irregular frequency spectra. Components obtained were compared to those reported earlier for validation. Only those corresponding in spatial distribution to components reported by Damoiseaux et al. were considered [26]. For the ensuing analysis we selected the topmost meaningful component, which represents the default mode network (i.e. component 4). Using the individual timecourses of the multi-session concatenated ICA components across subjects and conditions, we reconstructed subject-specific maps in native stereotaxic space using FMRIB's Improved Linear Model (FILM) in FMRI Expert Analysis Tool (FEAT). We then made group comparisons (using FMRIB's Local Analysis of Mixed Effects (FLAME)) to contrast the real and the 'sham' rTMS condition in a within-subject design; all results of the group analysis were warped to MNI standard space. We focused on the strongest meaningful component; this component corresponded to the default mode network (DMN).

BOLD signal contrasts for the comparison of the two groups were considered significant at a threshold of z > 3.1 (p < 0.001); to be sensitive to small but meaningful changes we conducted a directed search, i.e. within a mask consisting of the regions encompassed by the IC4.

## Abbreviations

BOLD: blood oxygen level dependent; DMN: default mode network; FMRI: functional magnetic resonance imaging; IC: independent component; PET: positron emission tomography; RSN: resting state network; (r)TMS: (repetitive) transcranial magnetic stimulation;

## Authors' contributions

YVDW designed the study, performed data acquisition and wrote the manuscript; EJSA supervised the analysis and co-wrote the manuscript; SM analyzed and interpreted the data; OAVDH designed the study, performed data acquisition and co-wrote the manuscript. All authors read and approved the final manuscript.

## References

[B1] Gusnard DA Akbudak E Shulman GL Raichle ME Medial prefrontal cortex and self-referential mental activity: relation to a default mode of brain function Proc Natl Acad Sci USA 2001 98 4259 4264 10.1073/pnas.071043098 11259662 PMC31213

[B2] Raichle ME MacLeod AM Snyder AZ Powers WJ Gusnard DA Shulman GL A default mode of brain function Proc Natl Acad Sci USA 2001 98 676 682 10.1073/pnas.98.2.676 11209064 PMC14647

[B3] Horovitz SG Braun AR Carr WS Picchioni D Balkin TJ Fukunaga M Duyn JH Decoupling of the brain's default mode network during deep sleep Proc Natl Acad Sci USA 2009 106 11376 11381 10.1073/pnas.0901435106 19549821 PMC2708777

[B4] Smith SM Fox PT Miller KL Glahn DC Fox PM Mackay CE Filippini N Watkins KE Toro R Laird AR Beckmann CF Correspondence of the brain's functional architecture during activation and rest Proc Natl Acad Sci USA 2009 106 13040 13045 10.1073/pnas.0905267106 19620724 PMC2722273

[B5] Eichele T Debener S Calhoun VD Specht K Engel AK Hugdahl K von Cramon DY Ullsperger M Prediction of human errors by maladaptive changes in event-related brain networks Proc Natl Acad Sci USA 2008 105 6173 6178 10.1073/pnas.0708965105 18427123 PMC2329680

[B6] Li CS Yan P Bergquist KL Sinha R Greater activation of the "default" brain regions predicts stop signal errors Neuroimage 2007 38 640 648 10.1016/j.neuroimage.2007.07.021 17884586 PMC2097963

[B7] Fox MD Snyder AZ Vincent JL Corbetta M Van Essen DC Raichle ME The human brain is intrinsically organized into dynamic, anticorrelated functional networks Proc Natl Acad Sci USA 2005 102 9673 9678 10.1073/pnas.0504136102 15976020 PMC1157105

[B8] Sridharan D Levitin DJ Menon V A critical role for the right fronto-insular cortex in switching between central-executive and default-mode networks Proc Natl Acad Sci USA 2008 105 12569 12574 10.1073/pnas.0800005105 18723676 PMC2527952

[B9] Kobayashi M Pascual-Leone A Transcranial magnetic stimulation in neurology Lancet Neurol 2003 2 145 156 10.1016/S1474-4422(03)00321-1 12849236

[B10] Chen R Studies of human motor physiology with transcranial magnetic stimulation Muscle Nerve Suppl 2000 9 S26 32 10.1002/1097-4598(2000)999:9<::AID-MUS6>3.0.CO;2-I 11135281

[B11] Fitzgerald PB Fountain S Daskalakis ZJ A comprehensive review of the effects of rTMS on motor cortical excitability and inhibition Clin Neurophysiol 2006 117 2584 2596 10.1016/j.clinph.2006.06.712 16890483

[B12] Chouinard PA Van Der Werf YD Leonard G Paus T Modulating neural networks with transcranial magnetic stimulation applied over the dorsal premotor and primary motor cortices J Neurophysiol 2003 90 1071 1083 10.1152/jn.01105.2002 12702714

[B13] Van Der Werf YD Paus T The neural response to transcranial magnetic stimulation of the human motor cortex. I. Intracortical and cortico-cortical contributions Exp Brain Res 2006 175 231 245 10.1007/s00221-006-0551-2 16783559

[B14] Ko JH Monchi O Ptito A Petrides M Strafella AP Repetitive Transcranial Magnetic Stimulation of Dorsolateral Prefrontal Cortex Affects Performance of the Wisconsin Card Sorting Task during Provision of Feedback Int J Biomed Imaging 2008 2008 143238 10.1155/2008/143238 18350118 PMC2266810

[B15] Strafella AP Paus T Fraraccio M Dagher A Striatal dopamine release induced by repetitive transcranial magnetic stimulation of the human motor cortex Brain 2003 126 2609 2615 10.1093/brain/awg268 12937078

[B16] Oliveri M Torriero S Koch G Salerno S Petrosini L Caltagirone C The role of transcranial magnetic stimulation in the study of cerebellar cognitive function Cerebellum 2007 6 95 101 10.1080/14734220701213421 17366271

[B17] Barrett J Della-Maggiore V Chouinard PA Paus T Mechanisms of action underlying the effect of repetitive transcranial magnetic stimulation on mood: behavioral and brain imaging studies Neuropsychopharmacology 2004 29 1172 1189 10.1038/sj.npp.1300411 15029151

[B18] Rounis E Stephan KE Lee L Siebner HR Pesenti A Friston KJ Rothwell JC Frackowiak RS Acute changes in frontoparietal activity after repetitive transcranial magnetic stimulation over the dorsolateral prefrontal cortex in a cued reaction time task J Neurosci 2006 26 9629 9638 10.1523/JNEUROSCI.2657-06.2006 16988033 PMC6674444

[B19] Rounis E Yarrow K Rothwell JC Effects of rTMS conditioning over the fronto-parietal network on motor versus visual attention J Cogn Neurosci 2007 19 513 524 10.1162/jocn.2007.19.3.513 17335398

[B20] Strafella AP Paus T Barrett J Dagher A Repetitive transcranial magnetic stimulation of the human prefrontal cortex induces dopamine release in the caudate nucleus J Neurosci 2001 21 RC157 11459878 10.1523/JNEUROSCI.21-15-j0003.2001 PMC6762641

[B21] Vanderhasselt MA De Raedt R Baeken C Leyman L Clerinx P D'Haenen H The influence of rTMS over the right dorsolateral prefrontal cortex on top-down attentional processes Brain Res 2007 1137 111 116 10.1016/j.brainres.2006.12.050 17229406

[B22] Vanderhasselt MA De Raedt R Baeken C Leyman L D'Haenen H The influence of rTMS over the right dorsolateral prefrontal cortex on intentional set switching Exp Brain Res 2006 172 561 565 10.1007/s00221-006-0540-5 16724174

[B23] Paus T Castro-Alamancos MA Petrides M Cortico-cortical connectivity of the human mid-dorsolateral frontal cortex and its modulation by repetitive transcranial magnetic stimulation Eur J Neurosci 2001 14 1405 1411 10.1046/j.0953-816x.2001.01757.x 11703468

[B24] O'Reardon JP Solvason HB Janicak PG Sampson S Isenberg KE Nahas Z McDonald WM Avery D Fitzgerald PB Loo C Efficacy and Safety of Transcranial Magnetic Stimulation in the Acute Treatment of Major Depression: A Multisite Randomized Controlled Trial Biol Psychiatry 2007 62 11 1208 16 10.1016/j.biopsych.2007.01.018 17573044

[B25] Helmich RC Siebner HR Bakker M Munchau A Bloem BR Repetitive transcranial magnetic stimulation to improve mood and motor function in Parkinson's disease J Neurol Sci 2006 248 84 96 10.1016/j.jns.2006.05.009 16793065

[B26] Damoiseaux JS Rombouts SA Barkhof F Scheltens P Stam CJ Smith SM Beckmann CF Consistent resting-state networks across healthy subjects Proc Natl Acad Sci USA 2006 103 13848 13853 10.1073/pnas.0601417103 16945915 PMC1564249

[B27] Fukunaga M Horovitz SG de Zwart JA van Gelderen P Balkin TJ Braun AR Duyn JH Metabolic origin of BOLD signal fluctuations in the absence of stimuli J Cereb Blood Flow Metab 2008 28 1377 1387 10.1038/jcbfm.2008.25 18382468

[B28] Larson-Prior LJ Zempel JM Nolan TS Prior FW Snyder AZ Raichle ME Cortical network functional connectivity in the descent to sleep Proc Natl Acad Sci USA 2009 106 4489 4494 10.1073/pnas.0900924106 19255447 PMC2657465

[B29] Damoiseaux JS Beckmann CF Arigita EJ Barkhof F Scheltens P Stam CJ Smith SM Rombouts SA Reduced resting-state brain activity in the "default network" in normal aging Cereb Cortex 2008 18 1856 1864 10.1093/cercor/bhm207 18063564

[B30] Filippini N MacIntosh BJ Hough MG Goodwin GM Frisoni GB Smith SM Matthews PM Beckmann CF Mackay CE Distinct patterns of brain activity in young carriers of the APOE-epsilon4 allele Proc Natl Acad Sci USA 2009 106 7209 7214 10.1073/pnas.0811879106 19357304 PMC2678478

[B31] Toro R Fox PT Paus T Functional coactivation map of the human brain Cereb Cortex 2008 18 2553 2559 10.1093/cercor/bhn014 18296434 PMC2567424

[B32] Jafri MJ Pearlson GD Stevens M Calhoun VD A method for functional network connectivity among spatially independent resting-state components in schizophrenia Neuroimage 2008 39 1666 1681 10.1016/j.neuroimage.2007.11.001 18082428 PMC3164840

[B33] Sack AT Cohen Kadosh R Schuhmann T Moerel M Walsh V Goebel R Optimizing functional accuracy of TMS in cognitive studies: a comparison of methods J Cogn Neurosci 2009 21 207 221 10.1162/jocn.2009.21126 18823235

[B34] Garrity AG Pearlson GD McKiernan K Lloyd D Kiehl KA Calhoun VD Aberrant "default mode" functional connectivity in schizophrenia Am J Psychiatry 2007 164 450 457 10.1176/appi.ajp.164.3.450 17329470

[B35] Kwon JS Jang JH Choi JS Kang DH Neuroimaging in obsessive-compulsive disorder Expert Rev Neurother 2009 9 255 269 10.1586/14737175.9.2.255 19210199

[B36] Sheline YI Barch DM Price JL Rundle MM Vaishnavi SN Snyder AZ Mintun MA Wang S Coalson RS Raichle ME The default mode network and self-referential processes in depression Proc Natl Acad Sci USA 2009 106 1942 1947 10.1073/pnas.0812686106 19171889 PMC2631078

[B37] Whitfield-Gabrieli S Thermenos HW Milanovic S Tsuang MT Faraone SV McCarley RW Shenton ME Green AI Nieto-Castanon A LaViolette P Hyperactivity and hyperconnectivity of the default network in schizophrenia and in first-degree relatives of persons with schizophrenia Proc Natl Acad Sci USA 2009 106 1279 1284 10.1073/pnas.0809141106 19164577 PMC2633557

[B38] George MS Nahas Z Molloy M Speer AM Oliver NC Li XB Arana GW Risch SC Ballenger JC A controlled trial of daily left prefrontal cortex TMS for treating depression Biol Psychiatry 2000 48 962 970 10.1016/S0006-3223(00)01048-9 11082469

[B39] Beckmann CF Smith SM Tensorial extensions of independent component analysis for multisubject FMRI analysis Neuroimage 2005 25 294 311 10.1016/j.neuroimage.2004.10.043 15734364

